# The behavioural state: critical observations on technocracy and psychocracy

**DOI:** 10.1007/s11077-018-9325-5

**Published:** 2018-06-18

**Authors:** Joram Nanne Pieter Feitsma

**Affiliations:** NWO TOP grant Welfare Improvement Through Nudging Knowledge, Utrecht University School of Governance, Bijlhouwerstraat 6, 3511 ZC Utrecht, The Netherlands

**Keywords:** Behavioural insights, Nudge, Technocracy, Psychocracy, Evidence-based policy, Dutch government, Ethnographic fieldwork

## Abstract

A ‘Behavioural Insights’ movement has emerged within governments. This movement infuses policymaking with behavioural scientific insights into the rationally bounded nature of human behaviour, hoping to make more effective and cost-efficient policies without being too obtrusive. Alongside sustained admirations of some, others see in Behavioural Insights the threatening revival of technocracy, and more particularly a ‘psychocracy’: a mode of public decision-making that wrongfully reduces the world of policymaking to a rational-instrumental and top-down affair dictated by psychological expertise. This article argues, however, that the claims of technocracy and psychocracy are overgeneralizations, emanating from a frontstage-focused debate that ignores a vast backwater of emerging behavioural policy practices. Grounded in four case studies on behavioural policymaking in Dutch governance, it will be demonstrated that at least part of this backwater is neither so technocratic nor so psychocratic as the critics claim.

## Introduction

A ‘Behavioural Insights’ movement has arisen within governments in the last decade. While this movement is still ‘under construction’, it has already become the object of a vast polemic debate, with some critics unmasking it as an updated form of technocracy with a psychological twist—i.e. a ‘psychocracy’. This article, however, argues that the portrayal of Behavioural Insights as technocratic and psychocratic is founded on only a handful of emblematic success stories which are not wholly representative for some of the alternative practices that have emerged in the ‘backwater’ of the Behavioural Insights field. I substantiate this argument with four case studies of not-so-technocratic and not-so-psychocratic behavioural practices, grounded in ethnographic fieldwork on upcoming behaviour experts in Dutch government.

The Behavioural Insights movement is part of a global policy agenda expressing a renewed interest in insights and methods from the behavioural sciences when underpinning and forming public policies (e.g. Strassheim and Korinek 2015; John 2018; Whitehead et al. 2017; Lunn 2012; Lourenço et al. 2016). An overarching behavioural insight is the idea that humans are not merely rational beings but have forgetful, lazy, ignorant, impulsive, and other less rational traits that make them behave against their own interests or goals. As the behavioural economics bestseller *Nudge* puts it: humans are more like Homer Simpson than *homo economicus* (Thaler and Sunstein 2008). The increased awareness hereof has resulted in the development of a more psychological style of governance that seeks to change behaviour by subtly redesigning local environments in ways that try to acknowledge and tap into the bounded rationality of policy subjects—all this with the support of rigorous experimental evaluation methods. This new style reflects in a changing professional apparatus of governments. Governments of today now inhabit ‘Choice Architects’, ‘Nudge Experts’, and ‘Chief Behavioural Officers’ (John 2018; Feitsma 2018). Perhaps the most vivid manifestation is the launch of ‘Behavioural Insight Teams’ (BITs): specialized units that are trialling novel types of ‘nudge’ interventions in a broad range of policy domains, from public health to tax compliance to sustainability. By now, there are BITs all across the globe, both inside and outside of government, from a ‘Behavioural Economics Team of the Australian Government’ to a ‘Qatar Behavioural Insights Unit’ to a ‘Behavioural Insights Network Netherlands’ (BIN NL). The initial trendsetter, the BIT in the UK, has grown from less than 10 to over 100 employees, with offices in London, New York, and Sydney, and has run over 600 Randomized Controlled Trials (RCTs). It has helped with, *inter alia,* promoting diversity in the police-corps, helping people to find a job faster, and getting people to pay their taxes on time (Dolan et al. 2010; BIT 2015). In the area of tax policy, for instance, it famously ran an experiment that increased tax return compliance with 5.1%, collecting an extra of GBP 9 million during a 23-day period, simply by including in the letters to late tax payers that ‘Nine out of ten people pay their tax on time. You are currently in the very small minority of people who have not paid us yet.’ (OECD 2017: 342). The British BIT has been one of the key missionaries in service of the ‘behavioural turn’ within governments, carefully constructing and promoting the Behavioural Insights ‘brand’ via a range of papers (e.g. Dolan et al. 2010; BIT 2012), update reports (e.g. BIT UK 2014; BIN NL 2017a) and books (Halpern 2015). Besides these specialized behavioural units, the behavioural turn is also boosted by the received support of distinguished politicians (e.g. David Cameron and Barack Obama), as well as the promoting work of influential transnational policy actors (e.g. the OECD, the World Bank, and the European Commission). Furthermore, behavioural scientists have increasingly been offering their expertise directly to governments. For instance, Richard Thaler, co-author of *Nudge* and recent Nobel Prize laureate, has been one of the key players in getting behavioural insights on the British policy agenda. Taken together, the new BITs and behaviour change professionals, along with their supporting top civil servants, think tanks, politicians, and academics, form a powerful frontstage chorus that speaks very positively about the emerging behavioural state, firmly believing that behavioural insights will help to make policies more effective and cost-efficient while still respectful of liberal freedoms (Dolan et al. 2010; Thaler and Sunstein 2008; World Bank 2015).

However, this celebratory if not self-advertising (Campbell 2017) chorus represents only one voice in the behavioural policy debate. Right from the beginning, and again particularly in the Anglo-Saxon sphere, also scepticism and criticism has been expressed against Behavioural Insights whether that be in public media, political or academic discourse. In the public media, the British BIT was welcomed with headliners as *Nudge Nudge, Say No More. Brits’ Minds Will Be Controlled Without Us Knowing It* (Website The Guardian 2014), and *Nudge* co-author Cass Sunstein was nominated as ‘the most dangerous man in America’ (Website Fox News 2010). In politics, the House of Lords expressed concern about the claimed added value of the British BIT, leading to an official investigation into the performance of this unit (House of Lords Science and Technology Select Committee 2011). In academia, many scholars have voiced critiques too. They have been sceptic about the added benefits of behavioural insights to policymaking and have also voiced concern about the underlying political and moral agenda of Behavioural Insights. So far, a range of normative critiques on the emerging behavioural state exist (Jones et al. 2013; Furedi 2011; McLaughlin 2016; White 2013; Whitehead et al. 2017; St. Paul 2011; Mulderrig 2017a, b; Mettler 2011; Leggett 2014; Rowson 2011; Campbell 2017). Initial concerns foresee the loss of liberal freedoms and autonomy, questioning whether citizens can direct their lives according to their own values when their everyday environments are increasingly supplemented with subtle governmental interventions. Further uneasiness is expressed with the idea of bureaucratic experts deciding what is good for citizens, possibly overriding their preferences (e.g. White 2013), leading particularly to the marginalization of lower-class income groups (Mulderrig 2017b). Underneath such concerns seems to lie a more generic concern about the possible upsurge of *technocracy.* By technocracy I mean a model of public decision-making in which bureaucrats decide *for* rather than *with* citizens, guided by scientific expertise rather than political dialogue (Clarence 2002). This ‘technocracy claim’ is partially due to that the nature of behavioural policy can be ‘stealthy’ (Whitehead et al. 2017; Mols et al. 2015). It can target and operate through the unconscious, automatic, *System I* (Kahneman 2011) decision-making faculties of citizens, and can take the form of pilot experiments that take place outside the public eye. This can make it rather difficult for citizens to notice or contest behavioural policies, let alone get involved in the decision-making process.

Disquietude over the resurgence of technocracy as the result of policy innovations is not new. The emergence of the policy analysis movement after World War II raised similar concerns (Fischer et al. 2015), just as, more recently, the rise of evidence-based policy (e.g. see Cabinet Office 1999) was explained as ‘technocracy reinvented’ (Clarence 2002). However, it should be noted that Behavioural Insights is associated with a particular technocratic form: a *psychocracy* (Jones et al. 2013). Psychocracy is characterized by its exclusive use of psychological knowledge and methods in the governance of citizen behaviours. This psychocratic nature of the behavioural state has also been criticized specifically (Mulderrig 2017a; Whitehead et al. 2017; Mols et al. 2015; Rowson 2011; Jones 2017; Feitsma 2018). One problem is that it has an overly psychologized idea of people’s ‘environments’, only looking at immediate, physical and technical aspects at the micro-level that have proved to affect decision-making in psychological experiments (Whitehead et al. 2017; Rowson 2011). This neglects that the environment is also shaped by deeper sociocultural, institutional, political, and economic forces at the macro-level that are much less ‘craftable’. Furthermore, criticisms have been voiced regarding the bias of the behavioural state towards a particular type of evidence, i.e. experimental knowledge (Feitsma 2018). Although such knowledge provides important information about ‘what worked there-and-then’ (Biesta 2007), policymaking would benefit from, if not necessitate inclusion of other types of valuable evidence (e.g. qualitative or experiential knowledge) in the policy process (Parsons 2002; Scott 1998; Fleming and Rhodes 2018). In sum, the ‘psychocracy claim’ holds that Behavioural Insights reduces the complex world of policymaking to a simplistic, monodisciplinary, and desocialized affair in which there is only space for the ideas and methods of psychological experts.

As its central empirical contribution, this article seeks to problematize the above-mentioned claims about the upsurge of technocracy in general—and psychocracy in particular—that are implied by the current behavioural policymaking trend. These claims are problematic because they emanate from a cemented ‘trench warfare’ type of debate in which participants speak only to and for their own ‘trenches’ (for an exception, see John 2017). This results in echo chambers that obstruct actual and nuanced debate. Moreover, the debate is muddled because its participants tend to make their arguments merely based on the frontstage manifestations and role models of Behavioural Insights. Antagonists have partly formulated their critiques based on abstract (dystopian) sketches about what a ‘nudge-world’ (Waldron 2014) might look like or on the iconic idea of behavioural policy as constructed by role models such as the British BIT*, Nudge,* and other published works (e.g. Dolan et al. 2010; BIT UK 2012, 2014; Halpern 2015). Because such role models tend to present generic ideas about how behavioural policy *should* be developed rather than how it actually *is* practised across various domains, these ideas do not necessarily offer an accurate and complete empirical basis for critical inspection.

In fact, in the debate’s focus on the frontstage facets of Behavioural Insights, a vast backwater of emerging behavioural practices is ignored. The Dutch government, for instance, is part of a glossed over hinterland, where nonetheless manifold behavioural practices have blossomed in the last decade (e.g. Schillemans and De Vries 2016). While there are some more substantial networks, such as BIN NL at national level and Behavioural Insights Group Rotterdam at municipal level, most of these practices are relatively small, informal, and in an experimental phase (Feitsma and Schillemans forthcoming). With the disregard of this backwater, the risk looms that an imprecise if not misrepresentative idea of behavioural policy is formed. To avoid this, this article sheds a new light on the behavioural policy debate—particularly with regard to the alleged upswing of technocracy—by shifting the current focus on the frontstage of Behavioural Insights over to some of the lesser-known backstage developments. I will zoom in on four different cases of behavioural policy practice, based on ethnographic fieldwork on behaviour experts in the Dutch government over a period of 4 years. By investigating these practices in relation to the themes of technocracy and psychocracy, I seek to stimulate critical thinking about the emerging behavioural state and expand it beyond the more well-known concerns about hampered autonomy and deepened paternalism (e.g. McLaughlin 2016; Jones et al. 2013). The key point, briefly put, is that the technocracy and psychocracy claims are overgeneralizations. The presented case studies show that behavioural practices can actually be quite democratic and inclusive of non-psychological expertise. While this does in no way make the critical claims irrelevant, it does suggest that they are at least unreflective of some developments in the field and the promise of a more ‘deliberative’ variant of behavioural public policy.

## Methods

### Design

This article takes off from an anthropological perspective that views the behavioural state not as a uniform, coherent, and abstract entity but rather as an assemblage of different, competing, and contradictory practices that operate both within but also further out of the deep state (Jones et al. 2013). From this perspective, the behavioural state is a peopled state, and it is these people that should be carefully studied. It is worthwhile to do ‘up close and personal’ ethnographic type of fieldwork (Rhodes et al. 2007) on these behaviour experts and their practices, to gain a deeper understanding of the behavioural state including its better hidden backstage realities (Van Hulst 2008). While a small body of qualitative studies on the behavioural state exists (e.g. John 2014; Jones et al. 2013; Whitehead et al. 2017), these studies remain largely grounded in generic and ‘thin’ empirical data, paying little attention to everyday experiences in the backstage.

### Data collection

This article is part of a larger ethnographic study on the rise of behaviour experts in government. For this study, I have interviewed, observed, and collaborated with behaviour experts in various levels of Dutch government for over a period of 4 years. The research has been undertaken in four research phases:

- From 2014 to 2016:
  - 24 interviews with 35 central governmental behaviour experts
  - 55 h of short-term participant-observation at various sites
  - Involvement as academic adviser in a local ‘Urban Nudging’ project over the course of 10 months
  - Document study
- In 2016:
  - 4 months of long-term participant-observation as employee-ethnographer in a small ministerial BIT, plus several pre- and post-visits to the field
  - Document study
- From 2016 to 2018:
  - 10 interviews with 15 local governmental behaviour experts
  - 19 h of short-term participant-observation at various sites of local governance, primarily municipalities but also a secondary school
  - Document study
- In 2018:
  - 1 focus group with a mixed group of governmental behaviour experts and academic experts

I started the research process with a mapping exercise of behaviour experts in Dutch government, based on an initial document study and the use of the snowballing technique. My sample only included self-identifying governmental behaviour experts who explicitly claimed to be making use of behavioural insights on a structural basis. My sampling strategy was partly pragmatic, doing participant-observations wherever I got access, and partly driven by the aim to capture the field of Dutch behavioural expertise in its full breadth. I ensured that my sample represented a broad range of public organizations, from ministerial departments to regulatory and executive agencies. Furthermore, I later conducted an extra round of ethnographic fieldwork, this time specifically zooming in on behaviour experts in local governments—which had up to then been a blind spot in my sample.

In terms of the conducted fieldwork, the first and third round of data gathering can best be described as a ‘yoyo’ type of fieldwork (Wulff 2002), making short visits to a broad range of behaviour experts in their professional habitats. Here, the focus was on doing semi-structured interviews. These were held in a closed setting, mostly where the interviewees worked, and guided by a protocol asking about general features of their work (e.g. organizational design, goals, tasks, everyday routines, successes, challenges) yet also leaving ample space to follow their own leads on where to take the conversation. The interviews were recorded (when having received permission to do so), selectively transcribed and turned into field reports. Next to interviews, I conducted various, mostly short-term participant-observations—such as shadowing behaviour experts for a day, attending internal work meetings, visiting educational meetings, having on-site informal conversations, and directly collaborating in projects. During or right after these observations, I jotted down quick notes, which I then translated into more elaborate field reports. The ‘yoyo’ type of approach initially helped to study the field of behaviour experts in all of its diversity. To achieve a deeper immersion in the field and see more of its backstage, I also collaborated more directly with behaviour experts, getting involved as an academic adviser in a local project that sought to incorporate behavioural insights. Moreover, as part of a second round of fieldwork, I was seconded to a Dutch BIT as an employee-ethnographer. Taking on the role of behaviour expert myself allowed me to develop a more tacit and experience-based understanding of behavioural policy practice. This whole trajectory of intensive and immersive fieldwork—going beyond the organizational frontstage, talking to and shadowing a multitude of experts, across various loci, over the course of years, while triangulating methods (i.e. interviews, participant-observation, document study) and organizing peer feedback—hopefully serves to bolster the trustworthiness and plausibility of my ‘conjectures’ (Rhodes 2014).

### Data analysis

The analytical process of my overarching study on behaviour experts also has a ‘yoyo’ element in it, constantly moving back and forth between doing fieldwork, ‘headwork’ and ‘textwork’ (Beuving and de Vries 2014; Bowen 2008). My earlier research projects were more oriented on *observing* and *describing* actual practices (see Feitsma 2018), professionalisms (see Feitsma and Schillemans forthcoming), and rationalities of behavioural policymaking. As the research progressed, and the iterative interplay between data collection and data analysis continued, my agenda also shifted to *debating* behavioural public policy. One particularly emerging topic for debate was that there seemed to be tensions between the rather polarized *Nudge* debate with its Anglo-Saxon focus and the lesser-known behavioural policy practices that I was studying. I sensed some discrepancy between what was happening within this backwater of behavioural public policy I was studying and how it was portrayed by some prominent *Nudge* critiques, claiming that behavioural policies are universally illegitimate, overpromising and ill-informed (e.g. Furedi 2011; Mols et al. 2015; St. Paul 2011). This article arose from this discrepancy. To do so, the analytical process for this particular study has been as follows. I started by breaking down the Behavioural Insights critiques based on a literature study. I then compared these with my fieldwork observations, and selected the two critical themes that most saliently seemed to point at a misfit between observed backwater practice and radical critique. I further worked out these themes, and their underlying assumptions (see Table 1 in the conclusion and discussion), and took them as sensitizing concepts to guide further empirical scrutiny. I then rescreened my data in light of these themes, looking for specific cases that made salient the misfit between the critical claims and my field observations. This screening followed a purposive selection strategy, searching for ‘information-rich cases’ (Bowen 2008) that best spoke to my sensitizing concepts and of which I had collected rich data from multiple sources—so as to increase trustworthiness. It needs to be emphasized that the case selection was based on the specific theory-driven question of whether counterexamples existed to the radicalizing technocracy and psychocracy critiques. I deliberately looked for counter cases, and my sampling strategy herein followed a logic of achieving theoretical adequacy—not a logic of achieving empirical representativeness. This means that the representativeness of my case studies should not be overstated (for further discussion on this, see the Discussion section). Nevertheless, putting the spotlights on such counter cases is important, given the overwhelming general academic consensus that behavioural public policy in its current stage of development indeed has a technocratic and psychocratic nature (John 2018). It may help to arrive at a more realistic, differentiated understanding of behavioural public policy.

The core analysis that follows consists of an analysis of four cases of lesser-known practices that take place in the backwater of Behavioural Insights. First, I explore two cases in relation to technocracy, and then, more specifically, two cases in relation to psychocracy. The case studies are introduced with a recap of the theoretical debate around these claims.

## Technocracy

The general critical claim investigated in this article concerns technocracy: a command-and-control model of public decision-making that is reliant on scientific expertise rather than political debate (Clarence 2002). A first sign of the technocratic character of Behavioural Insights could be derived from its basic ambition to infuse policymaking with (new) insights from the behavioural sciences so as to make policies more effective. This ambition thus combines a focus on the scientization of the policy process with an aim to optimize the effectiveness of policies. Implicitly, this shifts the focus away from more democratizing lines of inquiry, such as investigating current political needs or exploring how citizens can be involved, what knowledge they might have to offer, and what their preferences are.

The concern about the exclusion of citizens from the policy process particularly arises from the alleged ‘stealthy’ nature of behavioural policies (Whitehead et al. 2017; Mols et al. 2015; Mettler 2011). This stealthiness relates to the observation that behavioural policy tends to take shape in the form of rather subtle interventions, which in some cases deliberately target the unconscious decision-making faculties of citizens (although besides ‘exploitative’ there are also more ‘educative’ types of nudges, see Schubert 2017), and which sometimes affect citizens as part of undisclosed experiments. For instance, various Dutch government agencies have been making subtle changes in the letters that they send to policy subjects, inspired by the work of BIT UK in the context of tax policy (BIT UK 2014). Apart from the ‘Team Behaviour Change’ of the Dutch Tax and Customs Administration, which shared some of the results of its nudge-experiments in the public media (NRC 2015), there is little public information available about the exact nudge-interventions that these agencies have been using, nor about the letter experiments that they have been conducting. These stealthy aspects of behavioural policies make it difficult for citizens to observe, debate, reject, or give their informed consent to them. They hinder the formation of Habermasian ‘deliberative spaces’ in which behavioural governments are held accountable for their actions (Whitehead et al. 2017; McLaughlin 2016). Put differently, such developments may contribute to the rise of *the submerged state* (Mettler 2011), in which government actions increasingly become concealed to the general public, thereby hampering the possibilities of democratic decision-making and obfuscating the relationship between governments and citizens.

And yet, if we look at the actual behavioural policy practices that have emerged throughout governments, the portraiture of Behavioural Insights as technocratic seems somewhat problematic. I will mention some broad and preliminary observations. To begin with, ample attempts have been made to be transparent and create deliberative space about behavioural policy, for instance through various BIT update reports (e.g. BIT UK 2014), books (Halpern 2015), and scientific articles (John 2014). In the Dutch case, behavioural policymaking has also been subject to formal deliberation, with government advisory councils and behaviour experts organizing debates and writing reports about behavioural policy; one could even say there is more deliberative space than operational activity regarding behavioural insights in the Netherlands [e.g. BIN NL 2017a; WRR (The Netherlands Scientific Council for Government Policy) 2014]. The technocracy claim is furthermore challenged by the observation that the *targets* of behavioural policy are not per se citizens. In my interviews and participant-observations, I have found various cases in which other target groups were subjected to behaviour change tactics, such as commercial enterprises or policymakers. Moreover, it is also the case that the *makers* of behavioural policy are not per se governments. More often, these policies come about in wider fields of governance, in which governments collaborate with many other societal partners, playing a rather distanced—*meta*-*governing* (Sørensen and Torfing 2009)—role in the actual development and implementation of behaviour change interventions (Feitsma 2018). The observation that behavioural policies are neither per se made *for* citizens nor *by* government officials does not align with a technocratic frame of the state managing the citizen.

To give further empirical weight to the argument that the generalized technocracy claim is problematic, I will zoom in on two lesser-known cases of behavioural policy practice. These practices might even be considered to be democratizing, given their attention for the empowerment of citizens and public deliberation about new policymaking styles.

### Urban Nudging

Background characteristics

- Temporary project at local level
- Launched in 2015
- Collaboration between academics, artists, creative designers, and municipal officials
- Goal: facilitate societal debate about nudging

**Fig. 1:**
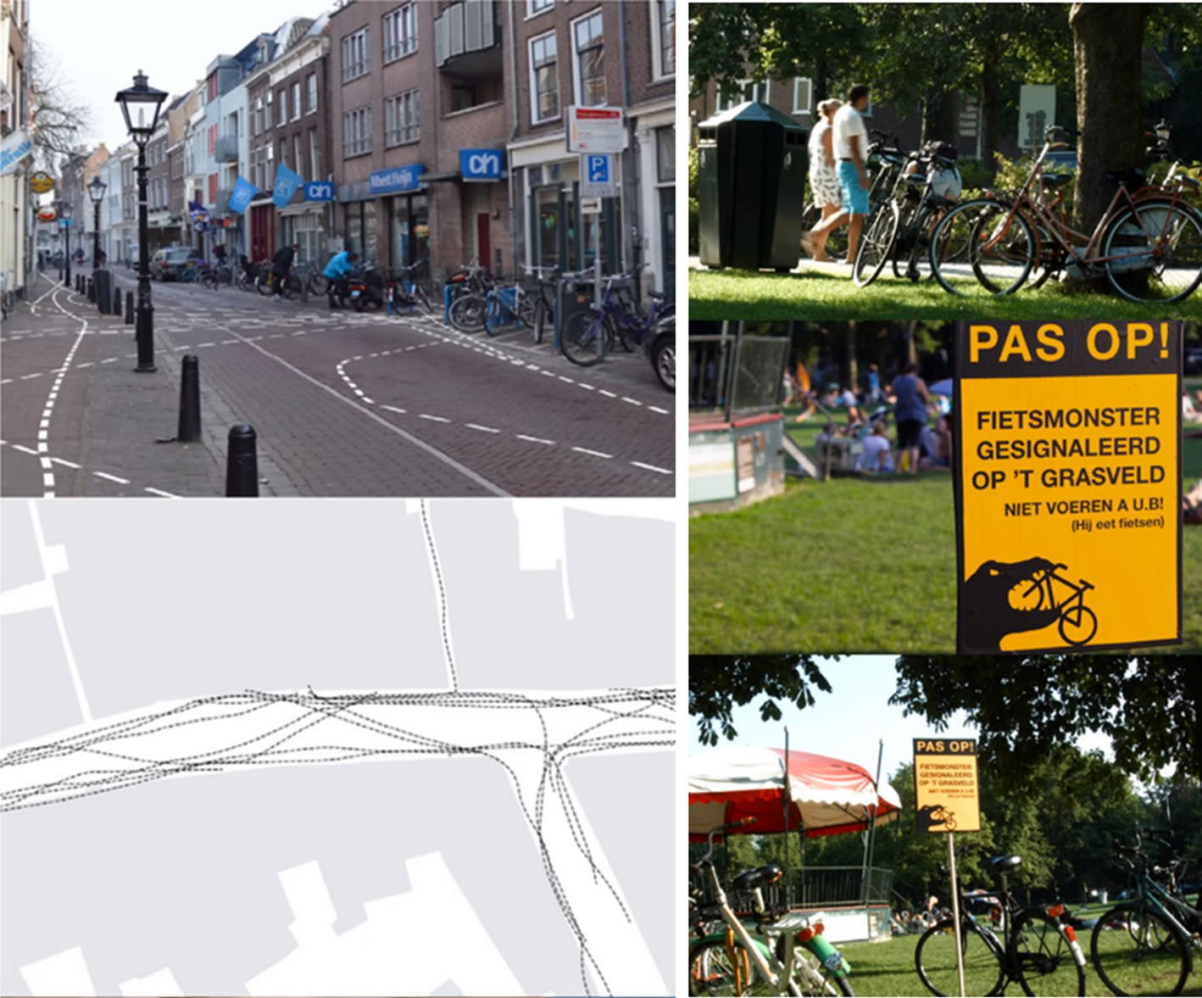
Urban Nudges

The first case-study features a rather atypical, small scale, and local project, called ‘Urban Nudging’, which was set up in the spring of 2015 in the Dutch city of Utrecht. This project was based on a collaboration between creative designers, artists, academics (including myself), and municipal public officials. The underlying goal of the project was to illustrate how behaviour change strategies could be developed in practice, so as to increase citizens’ own competencies in behaviour change. The specific challenge that Urban Nudging tackled was the wrong parking of bicycles in the city centre. This was a problem that the City of Utrecht had been struggling with for some time, as there were several ‘hotspots’ in the city centre where wrongly parked bicycles caused significant hindrance for pedestrians. After a short introduction into behavioural design and a field exploration, three different groups of artists and designers were deployed to design and realize their own nudges for the bike parking problem in the city centre, under the guidance of the project team and academic experts. The resulting interventions (see Fig. 1) were diverse in terms of their forms and the behavioural mechanisms that they sought to address. The more playful ones included a road sign warning bikers to watch out for a ‘bike monster eating up wrongly parked bikes’. A more sophisticated behavioural design was thought of by a group of landscape designers who analysed the various walking routes of pedestrians on a particular street, and then explored ways to visualize those routes, for instance by spraying traffic lines on the street, as a way of making bikers aware of other road users.

After having devised and implemented its behaviour change strategies for bike parking, Urban Nudging communicated its results to a broader audience of citizens in a project report (SETUP 2015). It developed an ‘Urban Nudging Kit’ that included the ‘ingredients’ of some of the developed nudges, meant to enthuse citizens to employ nudging techniques for themselves.

The lead in Urban Nudging was taken by SETUP, a small enterprise that focuses on societal innovations. SETUP launched this project out of an awareness of the popularity of novel behaviour change strategies. While it recognized the societal potential of these developments, it also noticed that these insights and behaviour change techniques were mostly taken up by businesses and governments. Citizens remained largely unaware of them. Believing that ‘knowledge about nudging should not only be reserved to a limited selection of people in power’ (SETUP 2015: 9), SETUP’s aim was thus to, in its own phrasing, ‘democratize’ nudging by introducing citizens into the world of behavioural insights.

In a series of playful nudges, we introduced the phenomenon in analogous forms to citizens of Utrecht. By stimulating them to make their own nudges, we have opened the debate about practices that are already influencing us on a daily basis, both offline and online. Urban Nudging is a democratization of the nudge; we celebrate the phenomenon of nudging, make these tricks accessible to everybody and activate citizens to think about the consequences, possibilities, and lastly the desirability of these practices. (SETUP 2015: 7–8)

This quote reveals an ambition to stimulate public deliberation about the use of behavioural insights. Urban Nudging tried to do so in a distinctive and interesting way. It sought not only to make fellow citizens aware of how they were continuously affected by public and private behaviour change strategies, but also aimed to facilitate them in using these strategies themselves. Hence, the project could be interpreted as empowering citizens in two distinct ways, both at odds with the technocracy critique. First, it aimed to increase their *behavioural literacy* (Whitehead et al. 2017) by making them more aware of everyday behaviour change efforts and the psychological mechanisms at work in these efforts. Second, the project aimed to develop the *behaviour change capacity* of citizens by showing how they themselves could harness the power of behavioural insights. This would enable them both to counteract behavioural influences imposed by external societal actors and also to ‘become part of an actual bottom-up problem-solving capital’ (SETUP 2015: 9).

Urban Nudging can be understood as an attempt to hold off a technocratic variant of behavioural policy. This case challenges the technocracy claim, first, by rejecting the assumption that behavioural policies are per se orchestrated by state officials. In Urban Nudging, state officials played only a marginal role. The lead was instead taken by a cultural enterprise which linked together actors from academia and the artistic domain. Furthermore, the idea that behavioural policies are accompanied by a lack of public deliberation and co-production is called into question. The central aim of Urban Nudging was to involve citizens in the behaviour change arena. Its efforts to build a decentred, bottom-up, collaborative behaviour change capital belie the claim that behavioural policies by necessity are produced and delivered in a command-and-control fashion.

### Litter-free communities

Background characteristics

- Structural, centrally orchestrated project
- Launched in 2017 but with earlier roots
- Broad, multi-level partnership with various government agencies and civil society actors
- Goal: develop effective anti-litter policy

While the previous case study featured a temporary project situated at the local level, in what follows a more structural project will be discussed, orchestrated by central government policymakers. In 2017, Rijkswaterstaat, the executive agency of the Dutch Ministry of Infrastructure and Water Management, collaborated with civil society actors to promote a national policy plan to reduce littering, called the ‘Landelijke Aanpak Zwerfafval (LAZ)’ [National Plan on Littering]. As an interviewed program coordinator of LAZ noted, this plan relied strongly on behavioural insights, particularly when it came to analysing which behavioural factors were at stake regarding the issue of littering. The program coordinator emphasized the importance of looking at unconscious and emotional aspects of decision-making and recognizing what he called ‘the power of the context’ in shaping human behaviour. *Prima facie,* LAZ could be deemed technocratic as in essence it sought to analyse and manage citizens from a distance with the help of science. This basic technocratic ambition reflected in the development of various anti-littering strategies on the basis of new behavioural insights (e.g. Novi Mores 2017).

What makes LAZ less technocratic, however, is its focus on participation and citizen activation. Based on the behavioural mechanisms behind perceived ownership and peer-to-peer influence, LAZ was underpinned by the belief that involving citizens in the decision-making process would yield more effective results. The perceived importance of citizen participation is exemplified by a collaboration of LAZ with a renowned behaviour change consultancy. This consultancy produced an advisory report on ‘Stimulating and maintaining participation regarding littering’ (Dijksterhuis and Van Baaren 2015), strategizing about how citizen participation could be facilitated, how participants could be kept ‘happy and active’, and also how municipalities should deal with initiatives from citizens. The report featured a menu with several strategies for municipalities to activate citizens. These strategies were thoroughly grounded in behavioural insights, suggesting subtle contextual readjustments that paid attention to the importance of symbolism, presentation, social norms, wording, and the framing and ordering of information. Suggestions included: making use of key active figures in the neighbourhood; using natural contact moments (e.g. local festivals) to communicate the desired behaviour; harnessing the power of reciprocity by handing out helpful tools (e.g. gripper waste pickup tools) as gifts; using communication signs that prime citizens with the idea of ‘togetherness’; turning communal cleaning events into pleasant experiences; using social rewards (e.g. sending out Christmas cards, thanking citizens for their contributions in keeping the community litter-free); and using the so-called ‘foot-in-the-door’ technique, first getting citizens to make a small commitment, after which to ask for a larger commitment which citizens will then be more likely to make for the sake of being consistent (Dijksterhuis and Van Baaren 2015).

The fact that LAZ sought to stimulate participation by hiring a consultancy firm specialized in behaviour change interestingly reflects the fusion of two seemingly conflicting policy strategies: the individual behaviour change focused ‘Nudge’ with the collective deliberation and participation oriented ‘Think’ (John et al. 2009). In LAZ, participation was viewed as a way of evoking behaviour change, and behaviour change strategies were used to stimulate and maintain participation. This interaction between ‘Nudge’ and ‘Think’ casts doubt upon the depiction of behavioural polices as purely technocratic. Rather, the less deliberative ‘Nudge’ tactics used in LAZ were used as second-order strategies in service of carrying out first-order strategies that sought to involve and activate citizens. On this first-order level, behavioural policies thus served a facilitating rather than impeding role in the cocreation of policies.

The fusion between ‘Nudge’ and ‘Think’ nevertheless raises questions about the assumed voluntariness and autonomy behind participation projects, and the desirability of governments undertaking concerted efforts to ‘nudge citizens into participation’. The LAZ case suggests that citizens’ choices to participate in public decision-making are not per se made wholly autonomously, but instead can be the result of a deliberately shaped process. This observation ties well with a Foucauldian perspective showing how late modern governments exercise power over citizens *through* freedom and self-regulation (Jones et al. 2013). In the LAZ case, what is at the surface perceived as citizens freely choosing to increase their control over their own communities, is actually impacted by subtle manifestations of governmental power that seek to orchestrate citizen behaviours through activation practices.

Besides its endeavours to involve citizens in the development of anti-litter policy, LAZ also sought to activate local government officials. To illustrate, an interviewed program coordinator noted that a substantial part of his work consisted in changing the behaviour of his peers in local governments, trying to involve them in the domains of littering and recycling policy. He framed his own challenges as follows: how could he get the issue of littering and recycling on top public servants’ policy agendas? And how might he persuade municipal officials to start using behavioural insights techniques within these policy domains? Recognizing the important role of municipal officials in realizing behaviour change, he sought to empower them with special guidelines, tools, training sessions, and long-term advisory trajectories educating them about behavioural insights. He also sought to motivate them using behaviourally informed strategies such as informing municipalities about their performance in relation to both the nationwide goals and the performance of other municipalities (i.e. giving feedback and communicating social norms). Also, he pressed top-level officials to write formal pacts as a sign of their engagement with the issue (i.e. leveraging the power of public commitment). A more sophisticated strategy was the development of a ‘Serious Litter Game’. This game presented involved policy actors with a fictional case in which they had to make policy on littering while taking on different stakeholder roles. This act of gamification was meant to put littering on the policy agenda in a ‘nice, relaxed, and effective way’, to stimulate an interactive and deeper learning process, and to have participants empathize with each other’s perspectives and ‘develop intuition about litter policy’ (Gemeente Schoon 2015). As these above-mentioned examples, and also the following quote, suggest, the program coordinator thus engaged with behavioural insights on a meta-perspective, seeking to change the behaviour of an intermediary group of policy officers who would on their turn then be better equipped to change the behaviour of citizens regarding littering and recycling:

How do I get the municipality to engage with this [policy]? That’s the first line of behaviour change that you’ve got in mind. So you’ve got to apply your knowledge also to persuade this target group. And the second line is: you want them to do it right, with a methodology, the right measures, the right consultancies, the right guidance. So we organize training programs and do benchmarking. (…) For instance, also in the program of household waste with regard to the role of municipalities, we applied A–M–O [ability–motivation–opportunity]. First we’ve got to motivate them and then we’ve got to give them the opportunities. If we can motivate management, resources will be made available, so then officials will get the opportunities to get started. In-between that lies ability: how do you make a proper policy proposal? Which measures do you use? How do you deal with residents? These sorts of things.

So you’re also nudging local policy officers?

Well, of course. I mean… you want to convince them… persuade them. How do you do that? There’s obviously a range of techniques that you can employ for that.

The focus in LAZ on activating local officials can be interpreted as another instance in which behaviour change strategies are fused with participatory aims. This particular case furthermore demonstrates that not only *citizens* but also *policymakers* are being subjected to behaviour change policies. This problematizes the technocracy claim which views the rise of Behavioural Insights in a rather one-sided way of how the state impacts its citizens, neglecting the behaviour change dynamics between the state centre (i.e. national government) and its peripheries (i.e. municipal governments) as illustrated in LAZ. When it is the state itself that is being affected by the behaviour change agenda, with behaviour experts practicing what they preach in their own policy networks, concern about the technocratic exclusion of citizens from the policy process seems less relevant.

## Psychocracy

From exploring the generic technocracy claim, the article now moves to investigating the more specific claim concerning *psychocracy:* a technocratic form that is specifically and exclusively grounded in psychology (Jones et al. 2013). This dominance of psychology relates both to the *ideas* that are included and the *methods* that are authorized as valid. With regard to both of these aspects, Behavioural Insights has been criticized (Mulderrig 2017a; Whitehead et al. 2017; Jones 2017; Feitsma 2018; Mols et al. 2015). On the level of ideas, the psychocracy claim holds that Behavioural Insights overly relies on psychological theories. Moreover, within this domain, it tends to focus exclusively on cognitive psychology, with less attention for the more social, motivational, and psychodynamic branches. A result of this is that the fields understand the ‘environment’ in a narrow, overly psychologized way (Whitehead et al. 2017). It thinks it can realize behaviour change simply by readjusting aspects of the immediate, physical, and technical environments of citizens—the so-called ‘choice architectures’ (Thaler and Sunstein 2008). Such an approach however fails to acknowledge the ‘obstinate nature of environmental legacies and the agency of the material world’ (Whitehead et al. 2017: 158). Environments are not as readily mouldable as Behavioural Insights claims. Moreover, environments do not simply consist of material objects, but rather of a complex amalgam of both material and immaterial objects shaped by micro-level effects but also by wider political, economic, sociocultural, and institutional forces (Shove et al. 2012). The disregard for such wider macro-level forces leads Behavioural Insights to believe that behaviour change can be ‘crafted’ through mere technical choice architecture. For instance, in the area of obesity policy, it may satisfice with subtly readjusting school canteen settings, rather than seeking to change the systemic economic inequalities that underpin unhealthy eating behaviours (Mulderrig 2017a).

On the level of methods, the psychocracy claim points at Behavioural Insights’ problematic understanding of ‘evidence’. Behavioural Insights’ underlying *evidence*-*based policy* ideology has been criticized for its rather peculiar understanding of the policy process and the role of evidence therein (Parsons 2002; Ingold and Monaghan 2016; Cairney 2017; Feitsma 2018). To begin with, Behavioural Insights thinks in a rather instrumental way about science-policy relation, as if academic knowledge flows naturally to the world policy, unaffected by political motives and interests. Such rational-instrumentalism however overlooks various by now well-documented observations of bounded rationality within the policy system, such as that policymakers tend to engage in satisficed decision-making based on incomplete analyses, dislike novel and complex, and maintain the status-quo or change it only incrementally (Lindblom 1959; Lodge and Wegrich 2016). Additionally, Behavioural Insights tends to overlook the political dimension of scientific knowledge (e.g. Ingold and Monaghan 2016; Weiss 1993; Cairney 2017). Many public administration studies have shown how evidence is handled, fabricated, selected, or shunned, in light of political interests that overshadow the instrumental function of science. Moreover, the particular hierarchy of evidence that Behavioural Insights promotes, with RCT-knowledge as the gold standard, is problematic. On a practical level, RCTs are only limitedly feasible as they are labour-intensive and technically complex methods, to be used in a politicized environment with scarce resources, bounded expertise, and a drive for swift decisions. On a methodological level, the claim that RCTs produce certain, fixed, universal knowledge is contestable (Whitehead et al. 2017). Rather than showing ‘what works everywhere-and-every-time’, RCTs provide provisional, isolated, ‘closed-system’ knowledge about ‘what worked there-and-then’ (Biesta 2007). Moreover, RCTs are only informative of the behavioural effects of a certain manipulation. They offer no understanding about why these effects happen, nor about how to actually use gained insights in unique local settings. Local knowledge, built up through practical experience over time, tends to be overlooked in Behavioural Insight discourse, because it cannot be easily explicated, codified, and disseminated as part of the body of behavioural insights (Parsons 2002). Behavioural Insights would nevertheless benefit from incorporating these non-experimental sources of evidence and horizontalizing its hierarchy of evidence (Rouw 2011).

However, as with the previous theme, the question is how universally valid the above-mentioned criticisms are in practice. Some initial observations at least partially belie them. To begin with, regarding Behavioural Insights’ supposed lack of attention for macro-level behavioural influences, a key publication in the field—*MINDSPACE—*already states that ‘sustainable changes in behaviour will come from the successful integration of cultural, regulatory and individual change’ (Dolan et al. 2010: 13), thereby showing an awareness of the multi-level complexity of policymaking. Furthermore, common tools in the field—like the ‘Gedragstoets’, the ‘Behaviour Change Wheel’, or the ‘Campaign Strategy Instrument’ (BIN NL 2017b)—also pay at least some attention to macro-level influences on behaviour in their promoted analytical frameworks. Second is the concern about Behavioural Insights’ narrow rational-instrumental understanding of evidence. At a basic level, my observations confirm the prevalence of such rational-instrumentalism. Dutch behaviour experts indeed tend to believe in the instrumental use of science and only scantily refer to the political function of evidence. In addition, the experimental method is celebrated widely in the field, and several behaviour experts appear to consider this method the most—if not only—valid way of gathering knowledge. But, in contrast with the critiques above, I also observed various behaviour experts who do not favour RCTs so exclusively, and draw from a wider range of sources of knowledge, including their own professional judgment and intuition.

To substantiate my argument that the claim of psychocracy is overstated, below I present two additional case studies of Dutch behavioural policy. The first case provides an example of how behaviour experts analyse policy problems from a wider, multidisciplinary angle. The second case shows that behaviour experts also draw from softer knowledge sources rather than just RCT-based knowledge, revealing the presence of a more pluralistic approach towards evidence use.

### Greening gardens

Background characteristics

- Temporary project at central level
- Launched in 2016
- Executed by a small team of policy advisers and academics
- Goal: explore behaviourally informed ways to increase the number of green (and thus more climate resilient) gardens in urban areas

This case-study features a temporary policy project that was launched around 2016 by the Dutch ministry of Infrastructure and Water Management. In this project, a new team of behaviour experts at the ministry explicitly sought to incorporate behavioural insights in the area of climate adaptation. The specific aim of this project was to stimulate citizens to make their gardens ‘greener’ as a way to improve the climate resiliency in urban areas. A ‘Climate Resilient Gardens’ project team was established that included internal policy advisers, scientists, and advisers from governmental knowledge institutes. This team followed an approach that was based explicitly on behavioural insights: the ‘DOE-MEE’ approach (see BIN NL 2017b). The team started by doing a ‘sanity check’ (see Hommes et al. 2016) in which it investigated whether this problem was a behavioural problem in the first place. When the sanity check confirmed the potential of a behaviour change approach, the team produced an in-depth behavioural analysis of why urban citizens would (or would not) invest in greener gardens (see Rietkerk et al. 2016). This analysis identified relevant behavioural determinants based on available scientific knowledge, while at the same time explicating what was still uncertain and inconclusive about this case. This meticulous and contextual way of working challenges the idea that Behavioural Insights practices automatically frame a certain issue as a behaviour change problem and then quickly move on to designing behavioural interventions. Rather, this team first performed detailed analysis on how much there really was to win with a behaviour change approach. Then it made extensive efforts to produce a behavioural analysis, while recognizing the provisional and inconclusive nature thereof.

Furthermore, it is interesting to shed light on the nature of the analysis that was produced in this project. This analysis was based on a scheme that distinguished between five factors: personal circumstances; abilities; motives; social environment; and decision-making processes (BIN NL 2017b). The first factor, personal circumstances, was said to relate to physical design but also to broader circumstances like the facilities, financial aspects, and law and regulations that surround people. This scheme helped to produce a fairly wide analysis. This analysis on one hand adopted the typical Behavioural Insights focus on micro-level decision-making processes. For instance, it recognized the socially contagious effects of ‘grey’ gardens which were increasing in numbers. Also, it addressed the fact that the impacts of a grey garden could be invisible to citizens; that grey gardens had short-term advantages over green gardens in terms of time and money investments; and that investing in green gardens might be hampered by ‘choice stress’ and a lack of ‘mental budget’ to perform garden activities. However, the analysis also addressed more systemic forces underpinning behaviour. For instance, it was found that wider social-cultural forces had a big impact on the choice for green gardens. People with, e.g. an older age, a female gender, a higher socio-economic status, a religion, and a western background, were more often found to have a green garden. Additionally, it was mentioned that it was important to look at the financial dimension behind this behaviour and the role of leisure time in today’s society, as grey gardens tend to be cheaper and easier to maintain.

The observed examination of citizen behaviour from both a micro- and macro-level perspective belies the critique that Behavioural Insights necessarily investigates behaviour from a psychological perspective, solely in relationship to the physical environment. In this case, the examination was implicitly underpinned by a more complex, historical, and institutional perspective on behaviour change. Even though this project was still partially grounded in psychology, and acknowledging the fact that making a wider macro-level analysis does not per se result in macro-level structural interventions beyond psychocratic individual behaviour change approaches, it at least demonstrated a clear awareness of the multi-level complexities of human behaviour and of the challenges involved in redirecting it.

### Living Nudge Lab

Background characteristics

- Temporary project at local level
- Launched in 2016
- Collaboration between schools, community centre, universities, and municipal officials
- Goal: experiment with public health-related behaviour change strategies in the real world.

In this case study, we make a shift from discussing the type of *ideas* that are prevalent in behavioural policy to the type of *methods* that prevail. Also, while the previous case plays out at the level of central government, the following is situated at the local level. It discusses a fairly atypical, small and temporary co-production between various local state and non-state actors. This collaboration—called the ‘Living Lab’—was launched in November 2016 in the Dutch city of Utrecht. Its specific goal was to experiment with public health-related behaviour change strategies in real-world settings. The collaborators in the Living Lab were a community centre, two secondary schools, the municipality of Utrecht, and a research group doing interdisciplinary nudge-research (of which I was part, although I was not directly involved in the execution of the research for the Living Lab project). The research took place in ‘Nieuw Welgelegen’, a large community building that hosted two secondary schools. The particular area of experimentation was the shared school canteen area in this building. The Living Lab ran trials of nudges directed at pupils in two specific areas of behaviour change. To stimulate pupils to stand (versus sit) during school breaks, standing tables were put in a more central place in the canteen. To stimulate pupils to drink more water (versus soft drinks), tap water points with fruit-flavoured water were placed in a prominent place. Besides measuring the effectiveness of these specific nudges, this project also explored the use of public health nudges from an ethical perspective. Surveys and focus groups were done to learn about how school pupils thought about being nudged into healthier behaviour. A general conclusion was that pupils felt that their health was important to them, and that although they were responsible for their health choices, they viewed nudging as a potentially helpful resource (Kroese 2017).

The conclusions from the nudge-experiments measuring effectiveness were more ambiguous. While the trial on standing behaviour during lunch breaks yielded no significant behaviour change results, these results were at the same time challenged by the researchers, for instance, by noting that some students also went outside during breaks and that those who stayed inside might already have a stronger urge to sit. The trial with the fruit-flavoured water taps also resulted in mixed observations. While a slight increase in self-reported water drinking was noted, the reliability of this result was called into question. For instance, it was noted that during the time of experimentation less students had been coming to the canteen due to outdoor school events. Also, a significant group of pupils which celebrated Ramadan had not been allowed to drink during school time at all for 1 week. Moreover, during the research process, it had not been feasible to conduct self-reporting surveys with the same group of pupils consistently. The awareness of such confounding variables all made it more difficult to draw hard conclusions. The summarizing research report thus repeatedly noted the difficulties of running trials in a dynamic field setting and interpreting the data generated from them. The report emphasized that these trials should be seen as pilots that provided lessons for future research projects that could then produce more robust results. Thus, the two clear-cut research questions that the project started with turned out to have more open-ended answers:

In this report, we address two questions: ‘is nudging effective’ and ‘is nudging acceptable’. It has to be noted that the research is embedded in the normal routines of the school program as much as possible, which makes the setting realistic but not ideal from a scientific perspective. The given conclusions will thus have to be viewed as lessons and ideas for the future instead of as hard evidence. (Kroese 2017: 5).

One can note several aspects about this Living Lab case that challenge the psychocracy claim that Behavioural Insights practices exclude valuable evidence from the policy process by prioritizing ‘what works’ knowledge. To begin with, while one part of the Living Lab indeed sought to experimentally assess the effectiveness of nudges, another part explicitly studied the ethical acceptability of nudges. This was accompanied by the introduction of specific methodological techniques (e.g. focus groups) and theoretical questions (e.g. political philosophical debates about state influence and citizen autonomy) that are allegedly less typical within behavioural policymaking. This widening focus may have been the result of the collaborative and multidisciplinary nature of the Living Lab—bringing together a range of actors representing a diversified set of interests, knowledge, and skills.

The Living Lab also seems reflective of the ultimately ambiguous nature of ‘what works’ knowledge. This is evidenced by the fact that the findings from both the standing table and the fruit-flavoured water tap trials were called into question by the research group and interpreted in an open-ended way. The research report repeatedly emphasized the uncertainty of the produced experimental knowledge and problematized the possibility of turning messy real-world sites into RCT-proof laboratory-like settings. The way in which the Living Lab challenged the possibilities of hard evidence-production nuances the wholly positivist image of Behavioural Insights as firm advocate of fixed, objective knowledge. At the same time, the research report stated that future experiments *would* be able to produce hard knowledge, suggesting that some of its positivist aspirations remained intact.

Another aspect that challenges the psychocracy claim relates to the effects of the Living Lab on the participating schools. One of the school principals noted during an interview that the project had led him to embrace behavioural insights in his daily work more generally. His uptake of behavioural science however seemed different than the rationalist version for which Behavioural Insights has been criticized. That is, he adopted a less scientific and more pragmatic approach, based on his own intuitive judgment on how basic behavioural theory might help to solve operational issues. He distanced himself from the demanding experimental ‘what works’ approach, and the necessity of having to collect hard scientific proof for his actions. When asked how he then knew whether his behaviour change strategies actually worked, he answered:

You don’t. That’s intuition. And I think that’s where it stops for us. I really mean that. I think that’s where it ends for us. (…) How great would it be if we’d know that the colour ‘red’ triggers people’s motivation to take the stairs, for instance? How great would that be? But in a different way, that’s also a utopia and you’d have to experimentally assess that. You’d really have to do it: the experiment. And on a small-scale that may be possible but on the large scale it’s mostly still about intuition though. (….) Maybe it’s the case that… you could do some literature study (…) but it remains a cost–benefit matter. (…) There are many other things that are very nice and interesting. And the benefit for me already is a change in my way-of-thinking. And that’s enough.

The principal’s view could be interpreted as revealing a shift from the exclusive appreciation of *episteme* to almost the exclusive appreciation of *phronesis* knowledge (Parsons 2002). As a self-proclaimed ‘believer in pragmatism’, the school principal practiced his own, adapted, experiential form of choice architecture: a form that was loosely informed by behavioural insights but beyond that underpinned by intuitive judgment, creative thinking, and satisficed decision-making. It is interesting to note the perceived split between academic behavioural practice and the everyday operational work. In the principal’s eyes, while scientific research may help to gain specific knowledge about the effects and working mechanisms of interventions, the idea of turning the school into a scientific laboratory is perceived to be, in his own words, a ‘utopia’. Scientific practice is regarded to be incompatible with his everyday work because he lacks the time and resources to go into theoretical detail or run experiments. Also, scientific study would in his eyes produce data that are less relevant at the operational level. The principal’s embrace of behavioural insights shows that this initially academic approach may be locally adapted into more experiential versions, suggesting that behavioural policymaking does not necessarily rationalize and psychologize the policy process as deeply as some critics claim.

Overall, the Living Lab forms a small scale but nonetheless interesting alternative case of behavioural policymaking based on a collaboration between actors in government, academia, and civil society. In this collaboration, different understandings of the role of evidence became apparent that challenged the alleged psychocratic nature of Behavioural Insights. The research group, while indeed prioritizing RCT-knowledge, nevertheless recognized the inconclusive nature of such knowledge and the methodological hardships of running experiments in real-world settings. An even more diverging understanding of the role of evidence was visible in the experiential choice architecture of the school principal, who relied on practical intuition rather than hard scientific knowledge.

## Conclusion and discussion

Contemporary governments have initiated a ‘behavioural turn’ in policymaking. This turn has had a polarized reception. Advocates point to the widespread failures of non-behaviourally informed policies in generating social change. Adversaries argue that the emerging behavioural state threatens to revive technocracy, and more particularly psychocracy, at the expense of citizen involvement and non-psychological sources of expertise. In its focus on frontstage developments in the field, the debate however overlooks a vast backwater of emerging Behavioural Insights practices. Grounded in ethnographic fieldwork over the course of 4 years, this article has therefore zoomed in on various lesser-known cases that have been developed in the peripheries of the field. A general conclusion is that behavioural public policy is more complex, fragmented and dynamic than the academic debate suggests. Neither the advocates nor the adversaries in this polemic debate have yet taken account of what the behavioural state currently *also* is. The advocates tend to disregard a range of critiques (concerning power, politics, and expertise) that operate underneath the current behavioural turn in policy. The adversaries tend to view the behavioural state as a coherent and finalized project, overlooking its diversity and dynamism. In addition, the radical tone of their critiques is at odds with the sometimes pastoral and incremental nature of behavioural public policy in its current form.

With regard to the claims of technocracy in general and psychocracy in particular, my conclusion would be that they are overgeneralizations. Four cases have been presented to reveal a more nuanced if not contradictory picture to some of the grand claims in the academic debate. Table 1 presents the specific empirical patterns reconstructed during the ethnographic fieldwork. To an important degree, it can be read as a concluding summary of the findings.

**Table 1:** Summary of empirical patterns

Theme	Theoretical claim	Empirical patterns	Examples from case studies
Technocracy	As behavioural policies are not easily observed by citizens, they are also rarely contested, debated and/or co-produced	I Behavioural policies can both be co-produced and be made subject of public deliberation II Behavioural policies can be used as second-order strategies to achieve first-order participatory goals III Behavioural policies can be targeted both at citizen and non-citizen actors	I Urban Nudging aimed to open-up the nudging debate, and empower citizens with behaviour change skills: *Urban Nudging is a democratization of the nudge* II Landelijke Aanpak Zwerfafval (LAZ) sought to ‘nudge citizens into participation’, exemplified by asking a behaviour change consultancy to conduct a behavioural study on *Stimulating and maintaining participation regarding littering* (Dijksterhuis and Van Baaren 2015) III LAZ also sought to activate municipal officials instead of citizens with regard to litter policy. *How do I get the municipality to engage with this [policy]? That’s the first line of behaviour change that you’ve got in mind*
Psychocracy	Behavioural policies seek to change behaviour through micro-level environmental redesign, and disregard powerful behavioural influences at the macro-level	Behavioural analyses can take into account macro-level behavioural influences	The Climate Resilient Garden project team conducted a wide behavioural analysis of garden-related behaviour of urban citizens. This analysis included macro-level circumstances like the facilities, financial aspects, cultural norms, and laws and regulations that surround citizens
	Behavioural policies are underpinned by a radical reliance on experimental knowledge, which is believed to provide fixed and universal knowledge. Other valuable methods of gathering evidence are excluded	I Behaviour experts can be aware of the ambiguities around evidence-based policy. II Behavioural policies can both be derived from experimental and non-experimental knowledge	I The Living Lab researchers repeatedly emphasized the inconclusive nature of their experiments in a setting that was *realistic but not ideal from a scientific perspective. The given conclusions will thus have to be viewed as lessons and ideas for the future instead of as hard evidence* II The school principal noted how the Living Lab had led him to incorporate behavioural insights, although in a more intuitive way. *How great would it be if we would know that the colour red triggers people’s motivation to take the stairs (…) But (…) that’s also a utopia and you’d have to experimentally assess that. (…) And on a small scale that may be possible but on the large scale it’s mostly still about intuition though*

Importantly, I am not dismissing present concerns about technocracy or psychocracy altogether. The ambition of this article was to show that these concerns do not apply *universally*. The presented case studies demonstrate that behavioural policies are neither intrinsically technocratic nor psychocratic. Rather, it is the producers of such policies who, in interaction with the wider institutional context, make such policies more (or less) technocratic and psychocratic. At the same time, the wider representativeness of the case studies in this article should not be overstated. The studied cases were purposively selected *because* they appeared to form counter cases to overgeneralizing claims. Also, the studied cases concern relatively small scale, often local and partially non-standard practices that take place in the context of Dutch governance. While Dutch behavioural practices to some extent mimic Anglo-Saxon Behavioural Insights role models, symbols and codes (Feitsma and Schillemans forthcoming), the Dutch context also comes with meaningful particularities that may shape local behavioural practice.

Moreover, the idea ought to be entertained that, rather than simply saying that the critical camp is too radical in its worries about the upsurge of technocracy and psychocracy, it actually may be the case that such worries have already (implicitly) impacted the development of behavioural policy at a deep level. As in a ‘self-denying prophecy’, the deliverers of behavioural policy may have sought to address the perceived risks and concerns voiced by the critics, leading to more moderated behavioural practices. My case studies to some extent confirm this idea. Urban Nudging, for instance, was launched as a *response* to the societally undebated ways in which behavioural science has been informing governments. And it is probable that the Living Nudge Lab research team explored the ethics of their behaviour change strategies *as the result of* an ongoing academic debate about the normative legitimacy of behavioural policies.

Whether the result of a self-denying prophecy or not, and while indeed still quite possibly odd cases in a more conservative landscape, the studied practices in this article at least reveal a potential of behavioural policies to withstand technocratic and psychocratic tendencies. They could be interpreted as attempts to develop a more *deliberative* type of behavioural public policy. Future research that further explores in which directions the global behavioural policy landscape is evolving, and whether a more deliberative trend is taking shape, is therefore worthwhile.
